# PReS-FINAL-2149: Interaction of PTPN2 and vitamin D GC sequence variants and risk of juvenile idiopathic arthritis

**DOI:** 10.1186/1546-0096-11-S2-P161

**Published:** 2013-12-05

**Authors:** JA Ellis, KJ Scurrah, RA Chavez, AL Ponsonby, A Pezic, RC Allen, JD Akikusa, JE Munro

**Affiliations:** 1 Genes, Environment & Complex Disease, Murdoch Childrens Research Institute, Parkville, Victoria, Australia; 2 Physiology, University of Melbourne, University of Melbourne, Australia; 3 Environmental & Genetic Epidemiology Research, Australia; 4 Arthritis Research, Murdoch Childrens Research Institute, Australia; 5 Paediatric Rheumatology Unit, Royal Children's Hospital, Parkville, Victoria, Australia

## Introduction

The causes of autoimmune diseases, including juvenile idiopathic arthritis (JIA) are presumed to involve the interplay of genes and environment. Vitamin D interacts with genes via vitamin D response elements (vdres). The gene *PTPN2*, associated with various autoimmune diseases including JIA, carries a VDRE and is regulated by vitamin D. Recently, an interaction between *PTPN2 *and *VDR *(vitamin D receptor gene) was reported for type 1 diabetes, such that disease association with *VDR *differed by *PTPN2 *genotype.

## Objectives

We aimed to detect interaction of *PTPN2 *with *VDR*, or with other vitamin D pathway genes (*GC*, *CYP2R1*, *DHCR7*, *CYP24A1*), in determining risk of JIA.

## Methods

15 snps were genotyped in 324 JIA cases and 568 controls from the childhood Arthritis Risk factor Identification study (CLARITY) in Melbourne Australia. We looked for association of each SNP with JIA in the entire dataset, and performed sensitivity analyses restricting to Caucasians (case n = 204, control n = 348) to control for population stratification. We then looked for interaction of *PTPN2 *with vitamin D genes in determining JIA risk on both a multiplicative and additive scale. Multiplicative interaction was assessed by the use of a product term in logistic regression. Additive interaction was assessed by calculating the Relative Excess Risk due to Interaction (RERI), along with 95% confidence intervals and p value using bootstrapping and permutation procedures respectively.

## Results

No snps were individually associated with JIA, except *GC *rs1155563 which was marginally associated in the entire dataset (p = 0.023), but not in Caucasians. A multiplicative interaction was observed between *PTPN2 *and *VDR *in the entire dataset (p = 0.016), but not in Caucasians. However, multiplicative interactions were observed between *PTPN2 *and *GC *which were evident overall and particularly among Caucasians (eg *PTPN2 *rs254151 and *GC *rs1155563: p = 0.0009). On an additive scale, possibly better reflecting biological interaction, we observed a RERI that deviated significantly from zero, providing further evidence of a negative interaction (*PTPN2 *rs254151 and *GC *rs1155563: RERI = -1.09; 95% CI -2.08, -0.38; p = 0.0004).

## Conclusion

We have identified interaction between *PTPN2 *and *GC *in determining risk of JIA in Caucasian children recruited to CLARITY. The evidence of interaction also on an additive scale adds biological plausibility. The data is consistent with the notion that *PTPN2 *is responsive to vitamin D, since GC variants alter the delivery of vitamin D to target cells, and rs1155563 is associated with circulating vitamin D levels. While our findings await replication, they do support the concept that vitamin D may be an environmental factor that interacts with immune-related genes in JIA.

## Disclosure of interest

None declared.

